# European survey on the use of patient contact shielding during radiological examinations

**DOI:** 10.1186/s13244-023-01452-3

**Published:** 2023-06-19

**Authors:** Claudio Granata, Erik Briers, Cristian Candela-Juan, John Damilakis, Timo De Bondt, Dario Faj, Shane Foley, Guy Frija, Hugo de las Heras Gala, Peter Hiles, Ruben Pauwels, Marta Sans Merce, Georgios Simantirakis, Eliseo Vano, Patrick Gilligan, the ESR, ESPR, EFOMP, EFRS, and EURADOS

**Affiliations:** 1 Department of Paediatric Radiology, Institute for Maternal and Child Health IRCCS Burlo Garofolo, Trieste, Italy; 2 Member ESR‑Patient Advisory Group, Patient Advocate, Hasselt, Belgium; 3 European Society of Radiology - EuroSafe Imaging, Vienna, Austria; 4 European Federation of Organizations for Medical Physics, Utrecht, The Netherlands; 5 Centro Nacional de Dosimetría (CND), Instituto Nacional de Gestión Sanitaria, Valencia, Spain; 6 University of Crete, Iraklion, Crete, Greece; 7 VITAZ, Department of medical physics, Moerlandstraat 1, 9100 Sint-Niklaas, Belgium; 8 AZ Sint-Blasius, Department of medical physics, Kroonveldlaan 50, 9200 Dendermonde, Belgium; 9 European Radiation Dosimetry Group, Neuherberg, Germany; 10 Faculty of Dental Medicine and Health, Osijek, Croatia; 11 European Federation of Radiographer Societies, Utrecht, Belgium; 12 Radiography and Diagnostic Imaging, University College Dublin, Dublin, Ireland; 13 Université de Paris, Paris, France; 14 Glan Clwyd Hospital, Bodelwyddan, Denbighshire UK; 15 Department of Dentistry and Oral Health, Aarhus University, Aarhus, Denmark; 16 Department of Radiology, Faculty of Dentistry, Chulalongkorn University, Bangkok, Thailand; 17 Geneva University Hospitals, Geneva, Switzerland; 18 Greek Atomic Energy Commission, Agia Paraskevi, Athens, Greece; 19 Radiology Department, Complutense University, Madrid, Spain; 20 Mater Misericordiae University Hospital, Eccles St., Dublin, Ireland

**Keywords:** Medical imaging, Radiation protection, Patient shielding, Surveys and questionnaires

## Abstract

**Objectives:**

Contact shielding (CS) of patients during X-ray studies has been used for decades to protect radiosensitive organs. This practice has not changed much despite increasing evidence that CS is not useful in many cases. The Gonad And Patient Shielding (GAPS) group—founded by representatives of the main European bodies involved in radiology—promoted this survey to assess the current practice of CS among European radiology departments and the attitude towards a non-shielding policy.

**Methods:**

Over a four-month period (15 May–15th September 2021) European Society of Radiology and European Society of Paediatric Radiology radiologist members were invited to respond to a web-based questionnaire consisting of 59 questions.

**Results:**

225 centres from 35 countries responded to this survey. CS was routinely applied in at least one radiological modality in 49.2% of centres performing studies in adults, 57.5% of centres performing studies in children, and 47.8% of centres performing studies on pregnant women. CS was most frequently used in conventional radiography, where the most frequently shielded organs were the gonads, followed by thyroid, female breasts, and eye lens. 83.6% respondents would follow European recommendations on the use of CS when provided by the main European bodies involved in radiology.

**Conclusions:**

This review shows that CS is still largely used across Europe. However, a non-shielding policy could be adopted in most departments if European professional societies provided recommendations. In this regard, a strong commitment by European and national professional societies to educate and inform practitioners, patients and carers is paramount.

**Clinical relevance statement:**

According to this survey expectations of patients and carers, and skepticism among professionals about the limited benefits of CS are the most important obstacles to the application of a no-shielding policy. A strong commitment from European and national professional societies to inform practitioners, patients and carers is fundamental.

**Graphical Abstract:**

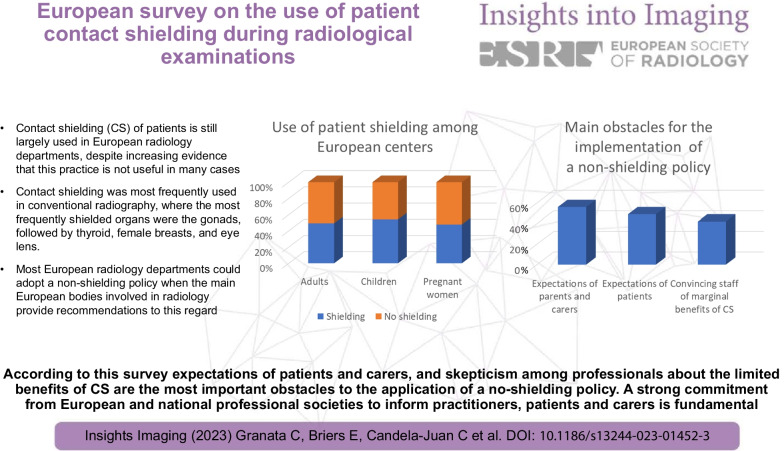

**Supplementary Information:**

The online version contains supplementary material available at 10.1186/s13244-023-01452-3.

## Key points

- Contact shielding of patients is still largely used in European radiology departments, despite increasing evidence that this practice is not useful in many cases.
- Contact shielding was most frequently used in conventional radiography, where the most frequently shielded organs were the gonads, followed by thyroid, female breasts, and eye lens.
- Most European radiology departments could adopt a non-shielding policy when the main European bodies involved in radiology provide recommendations to this regard.

**Supplementary Information:**

The online version contains supplementary material available at 10.1186/s13244-023-01452-3.

## Patient summary

Patient contact shielding (CS) has been used in radiology departments to “shield” patient organs from unwanted radiation doses, by application of a suitable radiation absorbing material. This practice is mostly used to shield radiosensitive organs such as the gonads, thyroid, eye lens, breast, and the embryo/foetus.

Recent advances in technology and evidence regarding the radiosensitivity of the organs in question have led to a new consensus on patient contact shielding. This European consensus is that contact shielding is no longer required for routine imaging.

This survey assessed the current use of contact shielding in Europe, the reasons why it happens or not, and the motivation to eventually follow the European consensus.

225 centres from 35 countries returned answers. Around half of the centres used CS in at least one radiological modality. These centres responded that CS “helps patients, carers, parents feel confident about the care received”.

Centres that did not use CS, are satisfied that CS is not effective.

Respondents pointed to patient and carer’s expectations, and difficulties convincing imaging staff as the main barriers to implementing new guidance.

The results from the survey point to CS still in regular use and that departments accept patients’ preferences as to the reason to do so, despite current guidance. Implementing the European Consensus will require an educational effort to inform and educate all involved professionals, patients and carers.

## Introduction

The application of patient contact shielding (CS) during X-ray studies has been in use for decades, with the aim to protect radiosensitive organs against unnecessary radiation [1]. The first application of shielding was to protect the gonads [2], although subsequently shielding was used to protect other sensitive organs such as the thyroid, breast, eye lens, and foetus in pregnant women [1].

The practice of shielding has not changed much despite increased knowledge on the effects of ionising radiation and advancements in X-ray equipment. Nowadays, improved collimation, tube shielding and beam filtration, better detector sensitivity, and Automatic Exposure Control (AEC) have led to significant reductions in patient adsorbed doses [3]. Additionally, the estimated radiation risk for various organs has changed over the years. In particular, the exposure of the gonads has always been of great concern, due to the supposed risk of inducing hereditary effects. However, recent genetic risk estimations in the human population have concluded that there is no evidence of a radiation-associated excess of heritable disease [4], and even data on foetal exposure suggest that the risk is small or non-existent at doses < 100 mGy [5]. Furthermore, the risk of missing diagnostic information due to a misplaced shield or of causing an increase in dose as a consequence of interference with AEC systems are well known issues [6].

Regarding these aspects, recent studies have raised concerns about the usefulness and effectiveness of shielding [3, 7, 8], adding to inconsistency in the regulation, recommendations and practice of shielding across Europe [9].

For these reasons, in 2020, representatives of the European Federation of Medical Physicists (EFOMP), European Federation of Radiographer Societies (EFRS), European Society of Radiology (ESR), ESR EuroSafe Imaging, European Society of Paediatric Radiology (ESPR), European Radiation Dosimetry Group (EURADOS) and European Academy of DentoMaxilloFacial Radiology (EADMFR), as well as a representative from the Patient Advisory Group of ESR, founded the Gonad And Patient Shielding (GAPS) group, to produce European Consensus recommendations on the use of contact shielding (CS) and to survey the current practice of patient shielding in Europe.

Based on the growing evidence provided in the recent literature [1–3, 5–8], the resulting European recommendations [10] concluded that in the majority of cases the practice of patient CS is no longer recommended, with the only exception of thyroid shielding, which may be considered when performing dental imaging.

In this article, we report the results of the survey that aimed to assess the current practice of patient CS among European radiology departments, and to investigate attitudes towards a non-shielding policy.

## Material and methods

### Ethical approval

Ethical approval was not required given the anonymised nature of the study, with no direct involvement of patients.

### Survey distribution

Over a four-month period (from 15th May to 15th September 2021) ESR and ESPR radiologist members were invited by the ESR EuroSafe Imaging office to respond in consultation with their head of Radiology Department and head radiographer to an anonymised, web-based questionnaire using Google forms. The survey included radiologists from countries of the European Union (EU), the European Economic Area (EEA), and from some non-EU/EEA countries.

### Survey questions

The survey was designed by the consensus view of all members of the GAPS group and endorsed by the ESR and ESPR boards. It consisted of 59 multiple-choice questions in the English language. A copy of the survey questions and response options are provided in the Additional file 1.

The questionnaire included both general and specific questions. The general questions aimed to assess the differences in CS practice across radiology departments in adults, children and pregnant women, and the reasons why CS was used or not used, awareness of recent recommendations issued by scientific bodies on the use of CS, and the possible issues related to the implementation of a non-shielding policy.

The specific questions were aimed to assess the use of shielding for certain radiologic studies, in selected group of patients and for specific organs. Multiple answers were possible for all of these questions.

### Data analysis

Data were analysed using descriptive statistics. Results were grouped according to use or non-use of CS, patients’ age (adults or children), and type of radiologic procedure performed.

## Results

### Demographics

20,604 radiologists were invited to participate, with responses received from 225 radiology departments. Responses came from 35 countries, with 168 (74.7%) respondents from EU/EEA countries and 57 (25.3%) from non-EU/EEA countries. A substantial proportion of responses (43.2%) were received from Italy (30, 13.3%), UK (22, 9.8%), Germany (17, 7.6%), Spain (16, 7.1%) and Turkey (12, 5.4%). The number of centres participating from each country is reported in Table 1.

**Table 1:** Participating countries, and number and percentage of participating radiology departments per country

EU/EEA countries	No. of answers	% of total respondents	Non-EU/EEA Countries	No. of answers	% of total respondents
Austria	3	1.3	Georgia	2	0.9
Belgium	9	4	Kazakhstan	3	1.3
Bulgaria	3	1.3	Montenegro	1	0.4
Croatia	1	0.4	Russian Fed	6	2.7
Cyprus	2	0.9	Serbia	1	0.4
Czech Rep	1	0.4	Switzerland	6	2.7
Denmark	2	0.9	Turkey	12	5.4
Estonia	3	1.3	Ukraine	4	1.8
Finland	10	4.4	UK	22	9.8
France	9	4			
Germany	17	7.6			
Greece	10	4.4			
Hungary	2	0.9			
Ireland	3	1.3			
Italy	30	13.3			
Latvia	3	1.3			
Lithuania	3	1.3			
Netherlands	7	3.1			
Norway	3	1.3			
Poland	5	2.2			
Portugal	8	3.6			
Romania	9	4			
Slovakia	1	0.4			
Slovenia	2	0.8			
Spain	16	7.1			
Sweden	6	2.7			
Total EU/EEA	168	74.7	Total NON-EU/EEA	57	25.3

### General questions

Of the 225 respondent centres, 193 (85.7%) performed radiologic studies in adults, of which 128 also in children, while 32 (14.3%) centres were dedicated paediatric radiology departments. Therefore, a total of 160 (71%) participating centres performed radiologic studies in children.

CS was routinely applied in at least one radiological modality in 95 centres (49.2%) out of the 193 centres performing studies in adults, 92 (57.5%) out of the 160 centres performing studies in children, and 54 (47.8%) out of 113 centres performing studies on pregnant women.

81 centres (36%) answered that a specific legislation or national recommendations about shielding existed for all organs and examinations, 76 (33.8%) for some organs and examinations only, and 34 (15.1%) answered that no recommendations or legislation were available; the answer was "not known" for 34 (15.1%) centres.

### Questions for the 146 centres using CS

The most frequent reasons for the use of CS were: "shielding is effective in reducing unnecessary dose exposure to sensitive organs" (97, 66.4%), "shielding helps patients, carers, parents feel confident about the care received (81, 55.5%), and "regulations require us to do so" (76, 52.1%).

When asked if at some stage they had experience of consequences from the use of CS, the most frequent answers were: "need to repeat the study due to superimposition of the shield" (107, 73.3%), "artefacts" (74, 50.4%), “increased dose due to automatic exposure control activation” (65, 44.5%), and “missed pathology” (51, 34.9%). 39 (26.7%) centres reported no consequences from the use of CS. Multiple answers were possible for both questions. Further details are available in Table 2.

**Table 2:** Questions for centres using and not using contact shielding: reasons why using and consequences of its use, reasons why not using and attitude about this policy

Centres using contact shielding	146	64.9%
Reasons why		
I believe shielding is effective to reduce unnecessary dose exposure to sensitive organs	97	66.4%
Shielding helps patients, carers, parents feel confident about the care received	81	55.5%
Regulations require us to do so	76	52.1%
Regulations require us to do so, but I do not believe shielding is effective or needed to reduce unnecessary dose exposure to sensitive organs	17	36.7%
Consequences of its use		
Need to repeat the study due to superimposition of the shield	107	73.3%
Artefacts	74	50.7%
Increased dose due to automatic exposure control activation	65	44.5%
Missed pathology	51	34.9%
None	39	26.7%
Infection control issues	8	5.5%
Centres not using contact shielding	79	35.1%
Reasons why		
It is because contact shielding is not effective or needed to reduce unnecessary dose exposure to sensitive organs	47	59.5%
It is because contact shielding may impair image quality and diagnostic capability of the examination – and therefore require retakes	46	58.2%
It is because the automatic exposure control (AEC) may increase the dose	41	51.9%
I don't know	12	15,2%
It is because of concerns for hygiene / infections	4	5,1%
It is because of the physical discomfort it brings to the patient	4	5,1%
Centres not using contact shielding	79	35.1%
How do you feel about this policy?		
I am OK with this policy	56	70.9%
I feel uneasy about this policy, as contact shielding may reduce unnecessary exposure to sensitive organs	10	12.7%
I feel uneasy with patients, as they may think we do not take care of them	8	10.1%
With children, I feel uneasy with their parents/carers, as they think we do not take care of them	5	6.3%
I don't know	4	5.1%
I feel uneasy with patients, as I am not able to explain effectively to them why they are not shielded	3	3.8%

Percentages are referred to the total number of centres using or not using contact shielding. Multiple answers were possible

### Questions for the 79 centres not using CS

When respondents from centres not using CS were asked for their reasons against CS use, the most frequent responses were: "because CS is not effective or needed to reduce unnecessary dose exposure to sensitive organs" (47, 59.5%), " because CS may impair image quality and diagnostic capability of the examination—and therefore require retakes" (46, 58.2%), and " because the AEC may increase the dose" (41, 51.9%). These centres were also asked how they felt about not using CS, with most (56, 71%) responding they were “ok with this policy". A small proportion expressed unease with the policy due to belief that CS may reduce unnecessary exposure to sensitive organs (10, 12.7%), or patients may think we do not take care of them" (8, 10.1%). Multiple answers were possible for both questions. More details are available in Table 2.

### Questions for all 225 centres

All participants in the survey were asked about the main challenges faced when discontinuing the practice of daily patient shielding. The most frequent answers were: "psychological aspects or expectations of parents/carers (127, 56.4%), patients" (111, 49.3%), and "convincing imaging staff of the marginal benefits/risks of shielding" (95, 42.2%).

Respondents were also asked if any patient/parent/carer complained if shielding was not used, with the most frequent answers being: "yes" (85, 37.7%), "no, even though we do not use CS routinely" (56, 24.9%), and "I don't know" (44, 19.6%). Multiple answers were possible for both questions and further more details are available in Table 3.

**Table 3:** Questions for all participating centres regarding the challenges when discontinuing the practice of CS in daily practice. Percentages are referred to the total number of centres

All centres	225	100%
When discontinuing the practice of shielding of patients in daily practice, what would be or what were the main challenges for implementation (tick all that apply)?		
Psychological aspects/expectations of parents/carers	127	56.4%
Psychological aspects/expectation of patients	111	49.3%
Convincing imaging staff of the marginal benefits/risks of shielding	95	42.2%
Lack of consistent messaging on the topic	70	31.1%
Personal education—I don’t fully understand why we should stop shielding and am not confident enough to inform others about the topic	66	29.3%
Did a patient/parent/caretaker complain if shielding was not used?		
Yes	85	37.7%
No, even though we do not use contact shielding routinely	56	24.9%
I don't know	44	19.6%
No, because we routinely use contact shielding	40	17.8%

Respondents were asked if they were aware of the position statements on the use of CS issued by the American Association of Physicists in Medicine (AAPM) [2] in 2019 and by the British Institute of Radiology (BIR) in 2020 [1]. Half of respondents (114, 50.7%) were aware of at least one of the two statements, whereas 111 (49.3%) were not.

Centres were then asked if they would follow European recommendations, if the main European bodies involved in radiology reached a consensus on CS in Europe, the majority (188, 83.6%) replied affirmatively, 28 (12.4%) did not know and 9 (4.0%) would not.

### Specific questions concerning the practice of CS

#### Conventional radiography

Information on CS practices was provided by 169 and 149 centres performing conventional radiology in adults and in children, respectively.

62 centres (36.7%) routinely used in-field shielding (IFS) in adults and 61 (40.9%) in children.

The most frequent in field shielded organs in adults and children were male gonads in pelvis radiography (46, 27.2% and 55, 36.9%, respectively), female gonads in pelvis radiography (37, 21.9% and 49, 32.9%, respectively), and female gonads in radiography of the spine (35, 20.7% and 38, 25.5%, respectively).

64 centres (37.9%) routinely used out of field shielding (OFS) in adults and 66 (44.3%) in children.

The most frequent OFS organs in adults and children were male gonads during abdominal X-ray (43, 25.4% and 53, 35.6%, respectively), female gonads during chest X-ray (42, 24.9% and 52, 34.9%, respectively), and male gonads during chest X-ray (41, 24.2% and 51, 34.2%, respectively). More details are available in the Additional file 1: Table S1.

### CT

154 centres performed CT in adults and 128 in children. 28 centres (18.2%) routinely used IFS in adults and 25 (19.5%) in children. The most frequent IFS organs were male gonads during pelvis CT (20, 13% in adults; 18, 14.1% in children), eye lens during head CT (12, 7.8% in adults; 13, 10.2% in children), and thyroid during neck CT (9, 5.8% in adults; 11, 8.6% in children). 37 centres (24%) routinely used OFS in adults and 40 (31.3%) in children. In adults the most frequent out of field shielded organs were female gonads during chest CT (24, 15.6%), male gonads during chest CT (23, 14.9%), and male gonads during abdominal CT (22, 14.3% in adults); in children, male gonads during extremity CT (34, 26.6%), female gonads during chest CT (33, 25.8%), and male gonads during chest CT (33, 25.8%). More details are available in the Additional file 1: Table S2.

### Interventional radiology procedures

120 centres performed interventional radiology procedures in adults and 83 in children.

36 of these centres (30%) routinely used OFS shielding in adults and 21 (25.3%) in children.

The most frequent out of field shielded organs were male gonads during abdominal procedures (24, 20% in adults; 21, 25.3% in children), female gonads during chest procedures (22, 18.3% in adults; 21, 25.3% in children), and male gonads during chest procedures (21, 17.5% in adults; 20, 24.1% in children). More details are available in the Additional file 1: Table S3.

### Dental imaging

83 centres performed dental imaging in adults and 74 in children. 23 centres (27.7%) routinely used OFS in adults and 23 (31.1%) in children. In adults the most frequent out of field shielded organs was the thyroid (panoramic radiography: 14, 16.9%, intraoral radiography: 11, 14.3%, and cone beam CT: 11, 13.3%; while in children the trunk (13, 17.6%), thyroid in intraoral radiography (11, 14.9%), and thyroid in panoramic radiography (11, 14.9%). More details are available in the Additional file 1: Table S4.

### Mammography

Of the 119 centres that performed mammography exams, 29 centres used OFS. The most commonly shielded organs were gonads (26, 21.8%), thyroid (14, 11.8%), and eye lens (4, 3.4%) (Additional file 1: Table S5).

### Radiologic imaging in pregnant women

113 centres performed radiologic imaging in pregnant women. Of these, 54 (47.8%) used OFS of the foetus. The most common studies in which shielding was used were chest radiography (50, 44.2%), chest CT (43, 38.1%), and extremity radiography (41, 36.3%). More details are available in the Additional file 1: Table S5.

## Discussion

This survey found that about half of centres routinely apply CS in at least one radiologic technique in adults and/or children. The main reason given for this was its effectiveness in protecting sensitive organs: this despite an increasing number of studies, position statements and recommendations that have raised concerns about its usefulness [1–3, 6–8]. Conversely, about one third of respondents using shielding do not believe in its effectiveness: this underlines that a common and international statement as that provided by the GAPS group [10] is strongly needed to support a homogeneous practice in the use of shielding.

More than half of the respondents using shielding answered that regulations required them to do so. However, a recent review by Candela-Juan et al. [9] showed that only a minority of countries have detailed regulations specifying when to use patient shielding. Most countries provide for its use when "necessary" or have no regulations at all, with professional societies providing guidelines or recommendations. Consequently, according to our results, knowledge of respondents on legal requirements of shielding appears to be poor.

Almost three-quarters of centres using CS reported adverse consequences from its use. The most frequent was the need to repeat the study due to superimposition of the shield on organs to be imaged, thus doubling the stochastic risk from the radiation exposure. Another common consequence was a shield impacting the operation of the AEC system when overlying the ionisation chamber. This leads to increased patient dose as the system delivers more exposure to account for the increased density and furthermore may be detrimental to the radiographic image quality. However, the potentially worst and—according to our results—not so uncommon effect of a misplaced shield can be misdiagnosis, when a lesion is hidden by a shield.

All these reasons argue in favour of the recommendations to carefully reconsider the use of shielding, as stated by the recent European Consensus Statement by the GAPS group [10].

On the other hand, about one-third of centres did not use CS, the main reasons being to avoid the above adverse consequences from CS use.

Almost three-quarters of respondents from centres with a no-shielding policy agreed with this policy, while the remainder felt uneasy, mostly because patients and carers could perceive non-shielding as a lack of care. On the other hand, about half of respondents from centres using CS believed shielding helps patients, parents and carers feel confident about the care received, as patients are used to getting shielding as a protection tool.

Indeed, very often, lay people perceive shielding as the only evidence of an attentive approach from the radiological team [1], so it is not surprising that about 40% of all participating centres referred to some level of complaints from patients not receiving shielding. Therefore, it is not unexpected that about 40% of all participating centres saw the psychological aspects and expectations of patients, parents and carers, and the lack of a consistent approach and specific education as the main challenges for the implementation of a no-shielding policy. Adopting a consistent approach, while simultaneously educating and informing patients/carers and health professionals of other more impactful optimisation measures such as collimation and individualised dose selection is crucial for this practice change.

While about half of respondents were not aware of the most recent recommendations issued by the AAPM and BIR on the practice of CS, the vast majority reported they would follow European recommendations when issued. This represents a strong stimulus for the relevant European professional bodies to ensure adequate education and training for imaging professionals and clear information for patients and carers are provided based on the recent European Consensus recently issued by the GAPS group [10].

As children are more radiosensitive than adults, it is not surprising that CS was most frequently used in children. Concerning the modalities, OFS was most frequently used (about 40% of centres) in adults and children for conventional radiography studies, followed by dental imaging (about 30% of centres). It is surprising to see that the use of OFS was more frequent in low dose procedures such as conventional radiography and dental imaging, although this could be explained by the long-standing habit of using CS in these modalities. Dental imaging is the one modality where the GAPS group considered shielding may be used in some circumstances to protect the thyroid due to lack of AEC use and the prevalence of larger collimator sizes on intra oral units. IFS was used in conventional and CT studies in about 40% and 20% of centres, respectively. This represents a worrying practice that should be stopped as it is not effective, may occlude important anatomical features and additionally interfere with AEC operation causing a dose increase if the shield impacts the AEC measurement [11, 12].

The most frequently shielded organs were the gonads, followed by thyroid, female breasts, and eye lens, again likely reflecting a legacy of the perception of high radiosensitivity of the gonads with the related genetic risks. However, heritable effects associated with the typical doses of diagnostic imaging are negligible [4]. Furthermore, the accuracy of shield application is very low, especially with ovaries, considering the variable position they may have within the abdomen [13].

OFS of pregnant women was used in about 50% of centres. However, it is well known that exposure of the foetus during studies not aimed to the abdomen does not represent a risk in most cases [5].

The education of professionals is pivotal when dealing with anxious patients such as pregnant women to reassure them and to concentrate on explaining the possible risks of shielding in addition to other more effective dose optimisation measures.

This survey has some limitations, in particular related to the low response rate. However, we did receive responses from a large number of countries, and believe our results are representative of current international practice*.* The paucity of responses however could also be taken as a sign of low interest among radiologists regarding the practice of CS. Another limitation of the study is that only a small number of dedicated paediatric radiology centres participated to the survey, making it difficult to compare practices in children with practices in general radiology departments.

This survey was targeted at radiologists. This could represent a possible limitation, as actually radiographers are in charge of the execution of the examinations and consequently are the most knowledgeable about their institutional policies and practices concerning CS. On the other hand, radiologists share with radiographers the responsibility of the best practice in terms of patient radiation protection and should be aware about CS practice. In any case, in order to mitigate this possible limitation, we recommended the radiologists to respond in consultation with their head radiographer.

## Conclusion

Information gathered with this survey show that CS is still largely used in European radiology departments, and especially with children. Psychological aspects in patients and carers, skepticism regarding the limited benefits of CS, poor knowledge and lack of consistent information appears to be the most important obstacles to the application of a no-shielding policy. However, most departments could adopt a no-shielding policy, if European professional societies provided recommendations. In this regard, the recent GAPS European consensus statement plays a pivotal role. A strong commitment from European and national professional societies to educate and inform practitioners, patients and carers is fundamental.

## Supplementary Information

**Additional file 1:** **Table S1.** Conventional Radiology: practice of in field and out of field contact shielding in adults and children, and organs shielded. Percentages are referred to the total number of centres performing conventional radiology. **Table S2.** CT: practice of in field and out of field contact shielding in adults and children, and organs shielded. Percentages are referred to the total number of centres performing CT studies. **Table S3**. Interventional Radiology: practice of out of field contact shielding in adults and children, and organs shielded. Percentages are referred to the total number of centres performing interventional radiology. IR: interventional radiology. **Table S4**. Dental Imaging: practice of out of field contact shielding in adults and children, and organs shielded. Percentages are referred to the total number of centres performing dental imaging. DI: dental imaging. **Table S5**. Mammography and radiologic imaging in pregnant women: practice of out of field contact shielding and organs shielded. Percentages are referred to the total number of centres performing mammography and radiologic imaging in pregnant women.

## Data Availability

Summary data from the survey is available in tables within the manuscript and Additional file 1. Upon reasonable request, further data can be made available from the corresponding author.

## Abbreviations

AAPM: American Association of Physicists in Medicine
AEC: Automatic Exposure Control
BIR: British Institute of Radiology
CS: Contact Shielding
EADMFR: European Academy of DentoMaxilloFacial Radiology
EEA: European Economic Area
EFOMP: European Federation of Organisations for Medical Physics
EFRS: European Federation of Radiographer Societies
ESPR: European Society of Paediatric Radiology
ESR: European Society of Radiology
EU: European Union
EURADOS: European Radiation Dosimetry Group
GAPS group: Gonads And Patient Shielding group
IFS: In Field Shielding
OFS: Out of Field Shielding
