# Supplementary material for: European survey on the use of patient contact shielding during radiological examinations
Source: Insights Imaging. 2023 Jun 19;14:108. doi: 10.1186/s13244-023-01452-3 (PMC10279619; doi:10.1186/s13244-023-01452-3)
Supplement: Supplementary file 1 — Additional file 1: Table S1. Conventional Radiology: practice of in field and out of field contact shielding in adults and children, and organs shielded. Percentages are referred to the total number of centres performing conventional radiology. Table S2. CT: practice of in field and out of field contact shielding in adults and children, and organs shielded. Percentages are referred to the total number of centres performing CT studies. Table S3. Interventional Radiology: practice of out of field contact shielding in adults and children, and organs shielded. Percentages are referred to the total number of centres performing interventional radiology. IR: interventional radiology. Table S4. Dental Imaging: practice of out of field contact shielding in adults and children, and organs shielded. Percentages are referred to the total number of centres performing dental imaging. DI: dental imaging. Table S5. Mammography and radiologic imaging in pregnant women: practice of out of field contact shielding and organs shielded. Percentages are referred to the total number of centres performing mammography and radiologic imaging in pregnant women. [file 13244_2023_1452_MOESM1_ESM.pdf]

ELECTRONIC SUPPLEMENTARY MATERIAL

European survey on the use of patient contact shielding during radiological examinations

Table S1. Conventional Radiology: practice of in field and out of field contact shielding in adults and children, and organs shielded. Percentages are referred to the total number of centres performing conventional radiology

**CENTERS PERFORMING CONVENTIONAL RX IN ADULTS**	**169**		**CENTERS PERFORMING CONVENTIONAL RX IN CHILDREN**	**149**	
**CENTERS USING IN FIELD SHIELDING**	**62**	**36.7%**	**CENTERS USING IN FIELD SHIELDING**	**61**	**40.9%**
**ORGAN SHIELDED**			**ORGAN SHIELDED**		
Male gonads in pelvis radiography	46	27,2%	Male gonads in pelvis radiography	55	36.9%
Female gonads in pelvis radiography	37	21.9%	Female gonads in pelvis radiography	49	32.9%
Female gonads in radiography of the spine	35	20.7%	Female gonads when performing abdominal X-ray	38	25.5%
Female gonads when performing abdominal X-ray	28	16.6%	Female gonads in radiography of the spine	34	22.8%
**CENTERS USING OUT OF FIELD SHIELDING**	**64**	**37.9%**	**CENTERS USING OUT OF FIELD SHIELDING**	**66**	**44.3%**
**ORGAN SHIELDED**			**ORGAN SHIELDED**		
Male gonads during abdominal X-ray	43	25.4%	Male gonads during abdominal X-ray	53	35.6%
Female gonads during chest X-ray	42	24.9%	Female gonads during chest X-ray	52	34.9%
Male gonads during chest X-ray	41	24.2%	Male gonads during radiography of the spine	51	34.2%
Male gonads during radiography of the spine	35	20.7%	Female gonads during chest X-ray	51	34.2%
Thyroid during skull radiography	27	16%	Thyroid during skull radiography	32	21.5%
Thyroid during chest radiography	17	10.1%	Thyroid during chest radiography	24	16.1%
Breast of females during skull or neck X-ray	15	8.9%	Breast of females during skull or neck X-ray	17	11.4%
Breast of females during abdomen-pelvis radiography	10	5.9%	Breast of females during abdomen-pelvis radiography	14	9.4%
Eye lenses during neck radiography	6	3.6%			

Table S2. CT: practice of in field and out of field contact shielding in adults and children, and organs shielded. Percentages are referred to the total number of centres performing CT studies

**CENTERS PERFORMING CT IN ADULTS**	**154**		**CENTERS PERFORMING CT IN CHILDREN**	**128**	
**CENTERS USING IN FIELD SHIELDING**	**28**	**18.2%**	**CENTERS USING IN FIELD SHIELDING**	**25**	**19.5%**
**ORGAN SHIELDED**			**ORGAN SHIELDED**		
Male gonads during pelvis CT	20	13%	Male gonads during pelvis CT	18	14.1%
Eye lenses during head CT	12	7.8%	Eye lenses during head CT	13	10.2%
Thyroid during neck CT	9	5.8%	Thyroid during neck CT	11	8.6%
Breast of females during neck CT	4	2.6%	Breast of females during chest CT	3	2.3%
**CENTERS USING OUT OF FIELD SHIELDING**	**37**	**24%**	**CENTERS USING OUT OF FIELD SHIELDING**	**40**	**31.3%**
**ORGAN SHIELDED**			**ORGAN SHIELDED**		
Female gonads during chest CT	24	15.6%	Male gonads during extremity CT	34	26.6%
Male gonads during chest CT	23	14.9%	Female gonads during chest CT	33	25.8%
Male gonads during abdominal CT	22	14.3%	Male gonads during chest CT	33	25.8%
Female gonads during extremity CT	19	12.3%	Female gonads during extremity CT	32	25%
Male gonads during extremity CT	18	11.7%	Male gonads during abdominal CT	29	22.7%
Breast of females in head or neck CT	15	9.7%	Thyroid during neck CT	18	14.1%
Thyroid during head CT	14	9.1%	Thyroid during chest CT	14	10.9%
Breast of females during abdomen-pelvis CT	11	7.1%	Breast of females during head CT	13	10.2%
Thyroid during chest CT	9	5.8%	Breast of females during abdominal CT	13	10.2%
Eye lenses during neck CT	8	5.2%	Eye lenses during neck CT	11	8.6%

Table S3. Interventional Radiology: practice of out of field contact shielding in adults and children, and organs shielded. Percentages are referred to the total number of centres performing interventional radiology. IR: interventional radiology

**CENTERS PERFORMING IR IN ADULTS**	**120**		**CENTERS PERFORMING IR IN CHILDREN**	**83**	
**CENTERS USING OUT OF FIELD SHIELDING**	**36**	**30%**	**CENTERS USING OUT OF FIELD SHIELDING**	**21**	**25.3%**
**ORGAN SHIELDED**			**ORGAN SHIELDED**		
Male gonads during abdominal IR	24	20%	Male gonads during extremity IR	21	25.3%
Female gonads during chest IR	22	18.3%	Female gonads during chest IR	21	25.3%
Female gonads during extremity IR	21	17.5%	Male gonads during chest IR	20	24.1%
Male gonads during chest IR	21	17.5%	Male gonads during abdominal IR	20	24.1%
Male gonads during extremity IR	21	17.5%	Female gonads during extremity IR	19	22.9%
Thyroid during brain IR	12	10%	Thyroid during chest IR	7	8.4%
Thyroid during chest IR	11	9.2%	Thyroid during brain IR	6	7.2%
Breast of females during brain IR	10	8.3%	Breast of females during brain or neck IR	5	6%
Breast of females during abdominal IR	10	8.3%	Breast of females during abdominal IR	4	4.8%
Eye lenses during neck IR	3	2.5%	Eye lenses during neck IR	2	2.4%
Eye lenses during chest IR	3	2.5%	Eye lenses during chest IR	2	2.4%

Table S4. Dental Imaging: practice of out of field contact shielding in adults and children, and organs shielded. Percentages are referred to the total number of centres performing dental imaging. DI: dental imaging

**CENTERS PERFORMING DI IN ADULTS**	**83**		**CENTERS PERFORMING DI IN CHILDREN**	74	
**CENTERS USING OUT OF FIELD SHIELDING**	**23**	**27.7%**	**CENTERS USING OUT OF FIELD SHIELDING**	23	31.1%
**ORGAN SHIELDED**			**ORGAN SHIELDED**		
Thyroid in panoramic radiography	14	16.9%	Apron on trunk	13	17.6%
Thyroid in intraoral radiography	11	13.3%	Thyroid in intraoral radiography	11	14.9%
Thyroid in cone beam CT	11	13.3%	Thyroid in panoramic radiography	11	14.9%
Apron on trunk	9	10.8%	Thyroid in cone beam CT	8	10.8%
Eye lenses in panoramic radiography	1	1.2%	Eye lenses in cephalometric radiography	1	1.4%
			Eye lenses in panoramic radiography	1	1.4%
			Eye lenses in cone beam CT	1	1.4%
			Gonads	1	1.4%

Table S5. Mammography and radiologic imaging in pregnant women: practice of out of field contact shielding and organs shielded. Percentages are referred to the total number of centres performing mammography and radiologic imaging in pregnant women

**CENTERS PERFORMING RADIOLOGIC EXAMINATIONS IN PREGNANT WOMEN**	**113**	
**CENTERS USING OUT OF FIELD SHIELDING OF THE FOETUS**	**54**	**47.8%**
Chest radiography	50	44.2%
Chest CT	43	38.1%
Extremity radiography	41	36.3%
Head or neck CT	39	34.5%
Extremity CT	38	33.6%
Skull or neck radiography	32	28.3%
Chest interventional radiology	29	25.7%
Skull or neck interventional radiology	27	23.9%
Abdominal CT (pelvis not included)	27	23.9%
Extremity interventional radiology	27	23.9%
Mammography	25	22.1%
Dental imaging	23	20.4%
**CENTERS PERFORMING MAMMOGRAPHY**	**119**	
**CENTERS USING OUT OF FIELD SHIELDING**	**29**	**24.4%**
**ORGAN SHIELDED**		
Gonads	26	21.8%
Thyroid	14	11.8%
Eye lenses	4	3.4%
